# Small Molecule Catalysts with Therapeutic Potential

**DOI:** 10.3390/molecules23040765

**Published:** 2018-03-27

**Authors:** Yannick Ney, Muhammad Jawad Nasim, Ammar Kharma, Lama A. Youssef, Claus Jacob

**Affiliations:** 1 Division of Bioorganic Chemistry, School of Pharmacy, Saarland University, D-66123 Saarbruecken, Germany; yannick.ney@uni-saarland.de (Y.N.); jawad.nasim@uni-saarland.de (M.J.N.); s8amkhar@stud.uni-saarland.de (A.K.); 2 Department of Pharmaceutics and Pharmaceutical Technology, School of Pharmacy, Damascus University, Damascus, Syria; ylama@hotmail.com

**Keywords:** catalysis, cellular thiolstat, drug design, enzymes, mimics, redox modulation, selenium, sensor/effector agents, transition metal complexes

## Abstract

Catalysts are employed in many areas of research and development where they combine high efficiency with often astonishing selectivity for their respective substrates. In biology, biocatalysts are omnipresent. Enzymes facilitate highly controlled, sophisticated cellular processes, such as metabolic conversions, sensing and signalling, and are prominent targets in drug development. In contrast, the therapeutic use of catalysts per se is still rather limited. Recent research has shown that small molecule catalytic agents able to modulate the redox state of the target cell bear considerable promise, particularly in the context of inflammatory and infectious diseases, stroke, ageing and even cancer. Rather than being “active” on their own in a more traditional sense, such agents develop their activity by initiating, promoting, enhancing or redirecting reactions between biomolecules already present in the cell, and their activity therefore depends critically on the predisposition of the target cell itself. Redox catalysts, for instance, preferably target cells with a distinct sensitivity towards changes in an already disturbed redox balance and/or increased levels of reactive oxygen species. Indeed, certain transition metal, chalcogen and quinone agents may activate an antioxidant response in normal cells whilst at the same time triggering apoptosis in cancer cells with a different pre-existing “biochemical redox signature” and closer to the internal redox threshold. In pharmacy, catalysts therefore stand out as promising lead structures, as sensor/effector agents which are highly effective, fairly selective, active in catalytic, i.e., often nanomolar concentrations and also very flexible in their structural design.

## 1. Introduction

**Figure 1:**
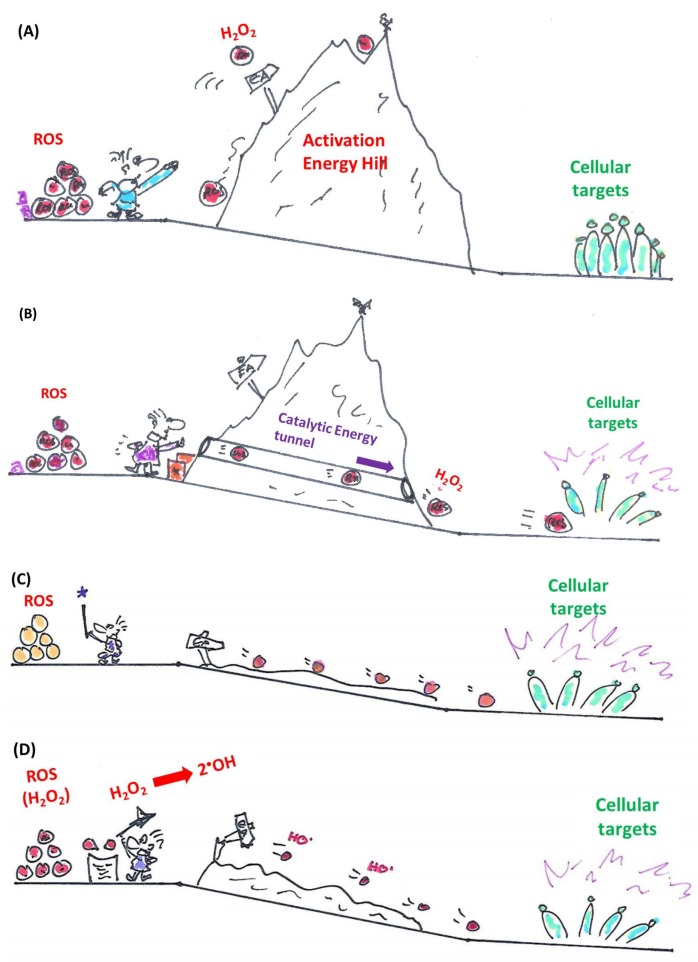
Cartoon illustrating the “catalytic principle” in the context of cellular processes. Inside cells various reactions are unfavourable kinetically and therefore possible yet slow (**A**). A suitable catalyst can accelerate such reactions significantly by “tunnelling through” the activation energy hill (**B**). It should be noted that this “catalytic tunnel” is not open to all kind of substrates, hence catalysts also provide a degree of selectivity (in this case for H_2_O_2_). Besides such direct catalytic interferences with cellular targets, certain catalysts are also able to deplete the cell of essential components, such as GSH or NAD(P)H or convert certain cellular components into natural or even unnatural, cytotoxic substances which react more rapidly with their cellular targets (**C**). A special and particularly attractive case is the interconversion of ROS (**D**).

Catalysts take centre stage in many scientific disciplines and processes, from large scale industrial manufacture of substances such as ammonia to the common exhaust of petrol-powered automobiles where platinum catalysts detoxify some of the toxic by-products of fuel combustion. According to Barack Obama, even civil society is “a catalyst for change”, yet chemistry employs a narrower definition of catalysis and catalysts involved [1,2]. Here, catalysts are agents which essentially promote the conversion of certain substrate(s) to product(s) by taking part in the reaction without being consumed or produced. Notably, the catalyst acts on the kinetics, not on the thermodynamics of the reaction. This means that: (a) its actions rely critically on the presence of suitable substrates and (b) catalysts may seemingly initiate, promote, enhance or even redirect energetically favourable reactions, yet are unable to trigger energetically unfavourable reactions. This aspect of catalysis is often overlooked and the cartoon in Figure 1 tries to emphasize this “catalytic principle”. In other words, although catalysts cannot perform thermodynamic miracles, they may speed up reactions which otherwise would be so slow that one may actually assume that they cannot occur at all. In addition, the catalyst is highly selective for the presence of suitable substrates and is limited in the range of products it is able to produce. Indeed, nothing happens if the catalyst is left devoid of its substrates.

**Figure 2:**
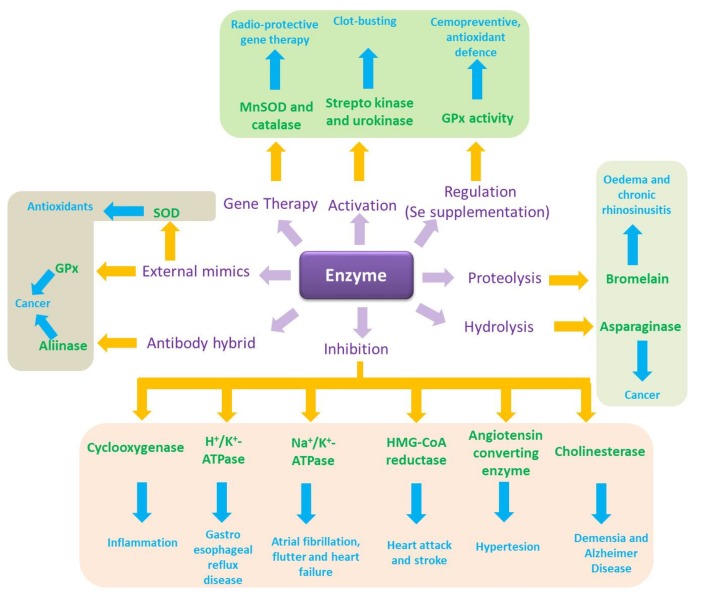
Enzymes take centre stage in innovative drug design, often as targets for inhibitors. See text for details.

Translated into biology terms, this simple notion of catalysis turns into the quintessence of cellular function, which is facilitated and controlled tightly by enzymes. It also provides an amazing spectrum of possibilities for intervention and, eventually, pharmaceutical drug design (Figure 2). Indeed, biology is an El Dorado for catalysis, where enzymes and other catalytic molecules, such as catalytic pieces of RNA, drive and control virtually all biochemical processes, from energy metabolism and cell cycle progression to redox signalling and apoptosis [3,4,5,6,7,8,9,10,11,12,13,14]. Enzymes are located intracellularly (i.e., in the cell membrane, cytosol, nucleus, mitochondria) or extracellularly and act in extremely small quantities. Such enzymatic action can exert a pronounced influence of the cell as enzymes are able to “process” hundreds or thousands of substrate molecules per second and some of them, such as superoxide dismutase (SOD), operate almost at the limit of diffusion with a catalytic rate constant of 1 × 10^−10^ M^−1^ s^−1^ estimated for human CuZnSOD and O_2_^●−^ at physiological pH [15].

Hardly surprising, pharmaceutical research for decades has considered enzymes, including human, bacterial, viral, fungal, and protozoal protein kinases, proteases, esterases, phosphatases and ATPases, as potential targets (Figure 2). Drugs containing enzyme inhibitors are legend, such as penicillins which inhibit a specific transpeptidase and serve as broad-spectrum antibiotics [16]. Such inhibitors are also prominent in other fields, for instance as antiviral drugs, where zidovudine and lamivudine inhibit nucleotide reverse transcriptase and serve in the treatment of human immunodeficiency virus (HIV), and in cancer research, where one only needs to consider methotrexate, which inhibits tetrahydrofolate dehydrogenase and hence impairs DNA synthesis and cell proliferation [17,18,19]. Along these very lines, a recent review by Stefania Schiavone and colleagues describes the role of various small molecule catalysts in neuropsychiatric and neurodegenerative disorders [20].

It is therefore startling that traditional drug design and development has consistently shied away from the wider therapeutic application of catalysts. Still, there are notable and highly instructive exemptions, examples of biological and chemical catalysts—in a wider sense—already employed or under development. As part of this review, and as prelude to the Special Issue on “Small molecule catalysts with therapeutic potential”, we will briefly consider some of these highlights with the aim to foster a wider understanding of their enormous potential and potential future applications.

## 2. The Use of Enzymes in Therapy

If one were to unleash the power of biocatalysis in therapy, the most obvious choice would probably involve the application of enzymes per se, as they are, literally, the natural choice of catalysts, being omnipresent in biological systems and evolved to highly efficient and at the same time selective macromolecules with impressive catalytic turnover rates and an amazing specificity for just one or a few substrates. Indeed, at closer inspection one finds some rather prominent examples of enzymes used already directly in therapy, often even by oral administration. A concise selection of these examples is mentioned in Figure 2.

The gastrointestinal tract, for instance, is a prime target for enzyme therapy, from a serious pancreatic enzyme replacement therapy for nutrient malabsorption (lipase, amylase, and protease) and pancreatic insufficiency to rather trivial yet notorious Beano^®^ anti-gas remedies based on the *Aspergillus niger* enzyme α-galactosidase [21,22,23]. Equally melodically and important are thrombolytic and fibrinolytic “clot-busting” agents which rely on a class of enzymes, originally streptokinase and urokinase [24]. These enzymes convert plasminogen to plasmin, which subsequently solubilizes fibrin and therefore degrades a blood thrombus and embolus. Even in the field of anticancer drugs, enzymes play some role. Asparaginase, a bacterial hydrolytic enzyme is used as an antineoplastic agent in humans as part of the chemotherapy regimen of acute lymphoblastic leukaemia (ALL), acute myeloid leukaemia (AML) and non-Hodgkin’s lymphoma [25,26,27]. Its mode of action is surprisingly simple yet effective. It hydrolyses asparagine to aspartic acid and hence deprives proliferating cells of this essential amino acid [25,28,29].

Another proteolytic group of enzymes relevant in human medicine is known as Bromelain, which actually stands for a mixture of cysteine proteases obtained from the stems, leaves and fruits of pineapple (*Ananas comosus*), and represents the active ingredient of a herbal remedy taken orally to treat oedema and chronic rhinosinusitis [30,31,32]. 

There are quite a few additional examples of enzymes serving as active ingredients—or even as preservatives—in medications and similar preparations. Still, the idea of using such macromolecules as drugs is somewhat daring, not because of lack of activity but due to an inherent, very poor pharmacokinetic profile. As for most proteins, oral availability is limited—although there are claims that enzymes such as the proteases of the Bromelian cocktail can be found in the bloodstream [32]. Unpleasant injections, usually intravenous, intramuscular or subcutaneous, remain the most common route for administering such therapeutics. Besides their poor oral bioavailability, enzymes are also prone to degradation, their transport into cells is complicated and, eventually, if injected, they may trigger a severe immune response, such as an extreme allergic reaction resulting in anaphylactic shock [33].

To circumvent such complications and issues with externally administered enzymes, different avenues have been explored, ranging from gene therapy to produce “therapeutic” enzymes at their desired destination and an induced (over-)expression of certain enzymes, to complicated antibody-enzyme hybrid systems and eventually small molecule enzyme mimics (Figure 2).

Gene therapy, which aims at the lifetime replacement of a defective gene with a corrected version, probably represents the most obvious but also revolutionary of these approaches. Strategies such as engineering SOD in cells under oxidative stress are quite convincing, and gene delivery of members of the SOD family, particularly manganese superoxide dismutase (MnSOD), and of catalase, are known as “radio-protective gene therapy” [34]. Today, clinical trials to test the safety and effectiveness of gene therapy for haemophilia A and B are ongoing [35]. Nonetheless, the successful implementation of gene therapy is extremely complicated in practice since it requires sophisticated delivery systems [36]. Vectors used to shuttle the corrected versions of the genes usually include plasmids, retroviruses, adenoviruses, and the adeno-associated virus (AAV) [37,38]. Even if a safe and effective gene therapy based on well-established vectors may spare the patients the agony of frequent enzyme drug injections, neutralizing antibodies, not only against the “healthy enzyme” but also against the—often viral—vector have been reported, further complicating this approach [39].

Less invasive alternatives are considering the controlled overexpression of enzymes from already existing genes. In fact, certain studies have demonstrated the prospect to increase the expression of enzymes by external stimulus, similar to the “induction” of protein expression in the liver or in bacterial cultures with the notorious isopropyl *β*-d-1-thiogalactopyranoside [40]. Glutathione peroxidase and thioredoxin reductase activity in the body can be increased, for instance, via an increased intake of dietary selenium [41,42,43]. Compared to gene therapy, this avenue towards increased amounts and activities of certain enzymes appears to be rather simple and mild, and is interesting, especially in the context of chemoprevention, food supplementation and quality of life in the elderly. Nonetheless, such primitive nutritional approaches are limited in scope. Concentrations of the relevant enzymes cannot be increased by large margins, the method is neither specific for just one desired enzyme or location, and in any case is limited to a handful of known examples. It is also somewhat difficult to control in practice, with over-supplementation of substances such as selenium representing a tangible risk.

Another alternative to the direct application of isolated enzymes involves prior modification to reduce the risk of being recognized as a target by the immune system. Eloquent enzyme-antibody hybrids, for instance, are not only able to “mask” an alien enzyme to prevent the immune response, but also to target the enzyme towards specific organs or cells. Here, cells interacting with the antibody of the hybrid via specific receptors appear to be affected preferably by this antibody targeted therapy, as has been demonstrated for the enzyme alliinase, which converts its harmless alliin substrate to the aggressive thiosulfinate allicin [44]. In a pioneering study by Rabinkov and colleagues, the intra-venous application of such an alliinase-antibody hybrid prior to the administration of alliin substrate via various routes, including orally, successfully targeted an ovarian carcinoma in a mouse model in vivo [45]. It appears that once injected into the bloodstream, the enzyme-antibody hybrid swiftly located at the preferred site of action and that the cytotoxic allicin was formed primarily at this site. Since allicin itself is not particularly stable chemically, similar studies have considered a combination of alliinase and synthetic alliin substrates as a cytotoxic “binary agent”, with possible applications in medicine and agriculture [46]. Related therapeutic “binary weapons” based on an enzyme and substrate combination include the enzyme myrosinase found in mustard and rapeseeds and its various glucosinolate substrates, which together form a spectrum of highly aggressive isothiocyanates, thiocyanates, nitriles and thiosulfinates [32,47,48].

Intriguingly, allicin by itself appears to be catalytic and since its discovery in 1944 by Cavallito and Bailey, has been studied extensively because of its pronounced cytotoxic activity [49,50]. As part of this Special Issue Slusarenko and his colleagues once more emphasize this unusual activity, whereby allicin is not only active against *Pseudomonas aeruginosa*, *Streptococcus pyogenes*, *Streptococcus agalactiae*, *Streptococcus dysgalactiae*, *Streptococcus pneumonia* or *Staphylococcus aureus* when added as liquid, but—astonishingly—even as a vapour [51]. From a pharmacological perspective, allicin may therefore bear some promise against lung pathogens, for instance via inhalation. In addition to “binary systems”, antimicrobial applications via the gas phase seem particularly attractive, as they avoid the kind of barriers faced along the oral route. As a sigh of relief, allicin and its decomposition products are usually “safe” for humans, as they are also formed within the human body from orally ingested garlic, and are even occasionally suspected of being active as bactericidal agents in the airways via the unique route of exhalation [51,52].

## 3. From Enzyme Mimics to Artificial Catalysts

Allicin serves as fragrant prelude to a wider range of natural and synthetic small molecule catalysts. Since most protein-based therapies, despite the various efforts described above, are still difficult to develop and to implement, chemists over the years have rather successfully explored “mimics” for a wide range of such enzymes, which primarily imitate their activity, yet avoid the “protein ballast” and complications which come with them. In essence, mimics retain certain pivotal features of the enzymes they are inspired by, for instance active site elements such as selenium, metal ions, functional groups or specific intermolecular interactions deemed important for activity and/or selectivity. Still, they differ otherwise dramatically from their parent enzymes, most obviously in size and chemical composition, but also in properties such as—metabolic—stability, polarity, solubility and bioavailability, all of which are important for possible therapeutic applications. It is a challenge for subsequent “generations” of mimics to “inch closer” to their respective parent enzymes, not only by higher catalytic turnover numbers but also by more predictable substrate profiles and other refinements which are often motivated by key features found in the parent enzyme and its active site. At the same time, synthetic chemistry is well equipped to deviate from the natural protein blueprint and to add specific design features, such an improved chemical stability and solubility, resilience against proteases and, eventually, desirable pharmacokinetic features along the “Lipinski in the Oral Office” catchphrase of 1990s drug design [53,54].

Glancing over the vast number of catalytically active mimics of different enzymes produced over the years, it is hardly surprising that some of them have also been considered as potential drugs, and redox modulating catalysts are no exception. Stimulated by the many transition metal based redox enzymes, numerous complexes of copper, iron, manganese, ruthenium, iridium and other transition metals have been designed, synthesized and evaluated extensively for biological activity. For instance, various mimics of the copper, zinc- and the manganese-containing superoxide dismutases, SOD1, SOD2 and SOD3, respectively, have been investigated as possible therapeutic antioxidants. Here, metalloporphyrin-based SOD mimics are the most widely known, yet other structures, such as Mn cyclic polyamines, Mn salen derivatives or even non-metal based molecules, among them water-soluble fullerenes, have proven their efficiency as good SOD-like catalysts [55,56]. Similarly, the family of antioxidant selenium-containing glutathione peroxidase (GPx) enzymes has given rise to a vast number of structurally highly diverse mimics which are generally based on selenium or tellurium and over the years have been considered as therapeutic antioxidants, but increasingly also as selective inducers of apoptosis (see Figure 2 and below) [57].

As in the case of SOD mimics, the chemistry at the active site of the parent enzyme may be seen as a guide but not as a must. Mugesh and his colleagues have recently described isonanoenzymes with a pronounced GPx-like activity and able to regulate the concentration of H_2_O_2_, yet based on V_2_O_5_, i.e., on a redox active element and material which differs entirely from the chalcogen-based small molecules traditionally considered in the context of GPx activity [58]. The fact that mimics—as small molecule catalysts—are considerably more flexible when compared to enzymes, and may also be incorporated into larger molecules, such as proteins and even nanomaterials, will be revisited later on.

Returning to catalysis and selenium, the compound ebselen (2-phenyl-1,2-benzoselenazol-3-one) has gained its prominence thanks to its catalytic activity and good pharmacological profile and ranks first among the very few selenium compounds which so far have entered clinical trials, in its specific case to prevent some of the damage caused by ischemic stroke and to treat symptoms of hypomania and mania in bipolar patients [59,60]. Although potential medical applications of ebselen are still debatable, this compound can be considered as a pioneer in the field of catalytic redox drugs.

Mimicking enzymes is therefore promising, but perhaps also narrows down a wider view on catalysis. Indeed, the desire to “inch closer” to the activity and substrate specificity of the parent enzyme, which for instance has long dominated the field of GPx mimic design, eclipses the rather interesting circumstance that more indiscriminate catalysts may actually open up new avenues. In the field of cytotoxic catalytic agents, where undesired or even alien reactions are employed to compromise the target cell, such a “badly behaved”, indiscriminate behaviour of the catalyst paradoxically may hold the key to selective therapeutic activity. Certain tellurium compounds support this notion. Originally developed as antioxidants, these agents have turned out to be more of a “loose redox catalytic cannon” in a cellular environment, and hence are able to target certain cells when employed in sub-micromolar concentrations [61,62]. In Group 16, the isosteric replacement along the line of sulphur—selenium—tellurium tends to enhance activity, and numerous tellurium compounds have been considered as potential antioxidants, not only in the context of human health and disease, but also for the preservation of materials and foodstuffs, as the research into radical-chain breaking agents by Engman and colleagues illustrates nicely [63,64]. Concurrently, moving from selenium to tellurium also diminishes any meaningful selectivity for GSH and turns the catalyst somewhat—but not entirely—indiscriminate against protein thiols (PrSH) (see Section 4). The resulting catalytic onslaught on the “cellular thiolstat”—a concept introduced to resolve the apparent paradox between indiscriminate chemical reactivity and pharmaceutical selectivity (see Section 4)—disrupts the intracellular redox homeostasis and hence renders many of these tellurium compounds outright toxic for certain types of cells [65]. Indeed, “loose cannon” redox catalysts with a certain selectivity for cells with an already disturbed redox signature have attracted quite some interest, not only in the context of GPx and chalcogens, but also in other quarters, which have, independently provided support for this simple yet elegant catalytic solution to efficiency and selectivity.

**Figure 3:**
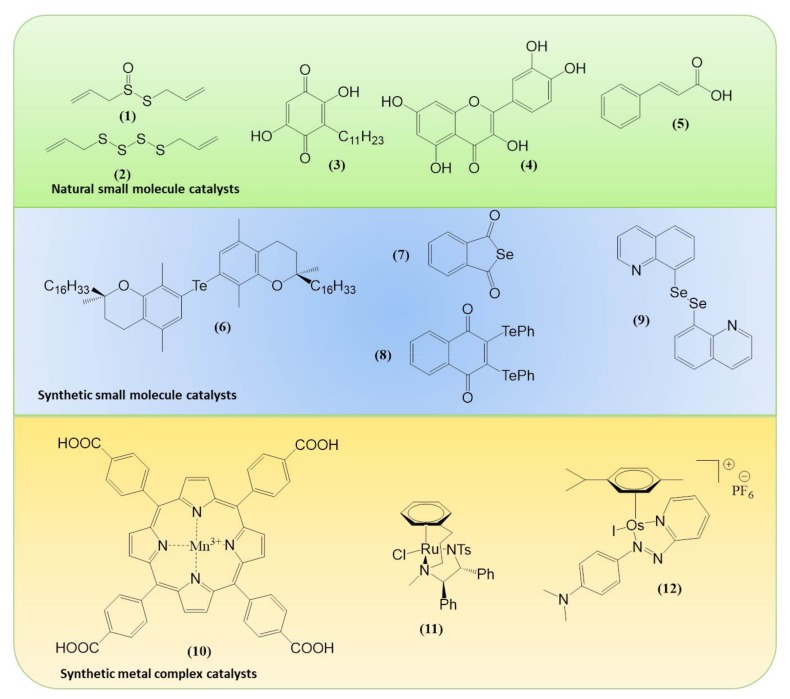
Chemical structures of the most important agents and catalysts discussed. allicin (**1**), diallyltetrasulfide (DATTS) (**2**), embelin (**3**), quercetin (**4**), cinnamic acid (**5**), 7,7′-tellurobis-*ß*-tocopherol (**6**), benzo[*c*]selenophene-1,3-dione (**7**), 2,3-*bis*(phenyltellanyl)naphthalene-1,4-dione (**8**), 1,2-di-(quinolin-8-yl)diselane (**9**), Mn(III)tetrakis(4-benzoic acid)porphyrin (**10**), (*R*,*R*)-[Ru(η^6^-C_6_H_5_(CH_2_)_3_Ts DPEN-*N*-Me)Cl] (**11**) and [Os(η^6^-*p*-cymene)(4-(2-pyridylazo)-*N*,*N-*dimethylaniline)I]PF_6_ (**12**).

**Figure 4:**
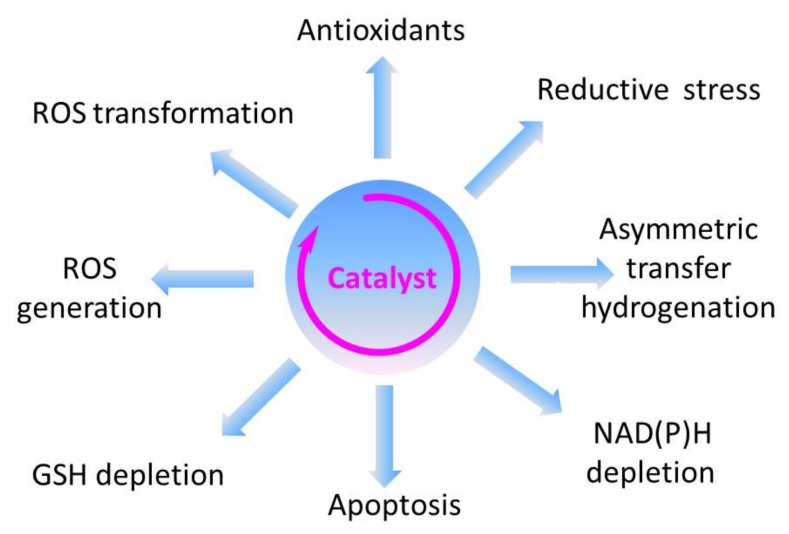
During the last decade, catalysts have been considered for various preventive and/or therapeutic purposes. Whilst natural agents with certain catalytic properties are de facto employed already as nutraceuticals in the field of nutrition or as nutrageceuticals (nutraceuticals employed to maintain or restore health and quality of life during ageing), other small molecule catalysts are still being developed and tested. Here, sensor/effector agents represent a particularly interesting class of agents as they fine-tune existing biochemical signatures. See text for details.

Within the realm of pharmacy some of the most striking recent examples of synthetic catalytic agents able to enter and impair living cells by an undesired and de facto detrimental catalysis involve organometallic compounds. Here, the Sadler group has been at the forefront of designing and developing transition metal-based catalysts able to perform unusual types of catalysis, often alien and damaging to certain types of cells (see Figure 1 for the catalytic principle and Figure 3 for relevant chemical structures). Playing virtuously and vigorously on the keyboard provided by the d-block elements in the Periodic Table, this group over the years has developed organoruthenium(II) as well as organoiridium(III) agents which catalyse various redox reactions, not only in vitro but also inside living cells. Some of the reactions facilitated involve the photochemical formation of singlet oxygen (^1^O_2_) by organoiridium(III) complexes in human lung cancer A549 and MRC-5 cells, ROS generation and depletion of reduced glutathione (GSH) in human ovarian A2780 and lung cancer A549 cells by Ru(II) complexes, as well as an interference with the central cellular NAD(P)H/NA(D)P^+^ redox couple in ovarian cancer A2780 cells by Ru(II) catalyzed Noyori-type transfer hydrogenation [66,67,68,69,70]. Very recently, a particularly interesting class of asymmetric transfer hydrogenation catalysts has emerged in the shape of Os(II) arene sulfonyl diamine complexes, which enter ovarian A2780 cancer cells where they reduce pyruvate in the presence of a suitable source of formate to either d- or l-lactate, whereby the stereochemistry of the product depends on the stereochemistry of the Os(II) complex applied [67,68,69]. In contrast to most of the previously employed small molecule redox catalysts, such as iridium, quinones, chalcogens, which act as oxidative stressors, these osmium-based catalysts cause a specific form of reductive stress. Since their catalytic activity relies on the presence of a suitable formate substrate, they also show some selectivity for ovarian cancer cells when compared to non-cancerous ovarian and lung fibroblast cells. One of these osmium complexes is also effective in attacking PC3 prostate cancer cells, albeit in this case rather unselectively, as its substrate *N*-formylmethionine is present in target PC3 prostate cancer cells as well as in normal cells [67]. The significance of small molecule catalysts in various fields is summarized in Figure 4. 

There are some lessons to be learned from the biological activity of these and similar small molecule redox catalysts. To achieve a more selective behaviour, one should avoid the need to administer both, catalyst and substrate, as this will diminish selectivity. One should also reconsider depriving cells generally of their fuel, such as NAD(P)H, GSH, or amino acids, such as asparagine and glutamine, as this will affect the cell in a wider, possibly uncontrolled manner [71]. In contrast, catalysts which operate with substrates limited to or primarily found in target cells or, alternatively, catalysts which generate products which are particularly damaging to the target cells, but not to normal cells, tend to attain higher selectivity. This fine but crucial difference between catalysts which rely on externally added substrates and eventually may result in “binary systems”, and catalysts which find and hence also sense the presence of their substrates in certain cell types is illustrated in Table 1. It should be noted that both types of catalysts have their own attraction, and both have been explored in the context of drug design, with different outcomes, strengths, drawbacks and potential applications.

## 4. Redox Catalysts with Sensor/Effector Properties

If one were to avoid the addition of extra substrates in combination with the catalyst, then small molecules able to transform reactive oxygen species (ROS) are a good choice. Firstly, ROS levels differ considerably between cells, and many target cells, such as microbes, inflammatory cells and cancer cells are particularly rich in ROS and hence also particularly prone to damage by ROS processing catalysts [72,73]. In fact, certain ROS can be converted catalytically into others (see also Figure 1), and inside cells, such conversions may dramatically increase the damaging properties of ROS, as exemplified by the transformation of hydrogen peroxide (H_2_O_2_) to the highly aggressive hydroxyl radical (^●^OH). Most of these ROS react with protein thiols as mentioned above.

Secondly, ROS are a fine choice of (pre- or pro-)cytotoxic agents as they tend to damage a wide range of biomolecules, yet in a controlled manner, which leads to cellular signalling and not simply to “poisoning”, as in the case of unnatural catalysts and products. Indeed, catalysts generating or employing ROS operate with “natural” oxidative stressors. Their impact on cells is therefore dramatic, yet paradoxically also controlled—often proceeding via distinctive signalling pathways and ending in programmed cell death, i.e., apoptosis. This implies that any catalyst promoting such damaging reactions would not only be highly effective, but also highly selective. It would act exclusively in the presence of ROS and cause damage via existing cellular pathways and without any major damage to normal cells or poisoning of the surrounding tissue and organism.

As prelude to this theme, and before considering individual small molecule catalysts in more detail, one may briefly turn towards the few cases where malfunctioning redox enzymes become associated with human disorders, as those natural examples highlight the severeness of catalysis gone badly. A mutated Cu, Zn-SOD in Familial Amyotrophic Lateral Sclerosis (FALS) is such an enzyme. FALS manifests itself in 5% of the patients with the motor neuron disease amyotrophic lateral sclerosis (ALS) [74,75]. From a molecular perspective, the deficient SOD is still active catalytically, yet has lost some of its specificity and besides converting O_2_^•−^ to H_2_O_2_ and O_2_ also reduces H_2_O_2_ to ^●^OH radicals. These radicals are amongst the most aggressive oxidative stressors and cause considerable damage to the cells and organs affected in patients suffering from this genetic disorder [76].

Perhaps in part inspired by this kind of “almost but not quite natural” cytotoxic redox catalysis, compounds such as the manganese complex mangafodipir (2-[2-[carboxylatomethyl-[[5-[[hydroxy(oxido)phosphoryl]oxymethyl]-2-methyl-3-oxidopyridin-4-yl]methyl]amino]ethyl-[[2-methyl-3-oxido-5-(phosphonooxymethyl)pyridin-4-yl]methyl]amino] acetate; manganese(2+)) have been applied successfully by Batteux and his colleagues as radical generators in a range of target cells, including human leukocytes and murine CT26 colon cancer cells [77,78]. Similar studies have confirmed these findings, employing, for instance, the superoxide dismutase mimic GC4419 to transfer one electron from ascorbate to O_2_^•−^, thereby subsequently generating the more aggressive H_2_O_2_ and distorting the intracellular redox balance [79]. Ironically, other SOD mimetics, such as MnDPDP and calmangafodipin [Ca_4_Mn(DPDP)_5_] have been described as selective cyto-protective agents, in essence acting via an antioxidant mechanism [80].

Whilst belonging to the same family of Mn-complexes, this apparent contrast in activity is due to the individual redox behaviour of the catalysts, the redox environment they are placed in, the kind of catalysis they perform, and the competition of simultaneously triggered, yet partially conflicting signalling pathways (see Section 5). In the case of mangafodipir, it is therefore conceivable that such agents selectively protect normal tissues from chemotherapy-induced toxicity, whilst they allow or even enhance damage to tumour tissue(s), for instance as part of a combination therapy with oxaliplatin [81]. In fact, a recent report on manganese porphyrins has stressed this dichtometry, whereby the same compound protects normal prostate cells against radiation damage whilst it inhibits the growth of prostate cancer cells [82].

By radical generation, and similar to the mutant SOD in FALS, such Mn-complexes do not interfere with their eventual cellular targets, such as redox-sensitive proteins, directly. These targets only become damaged once ROS are (trans-)formed and react further, or, in the case of protective agents, are guarded from oxidation because the relevant ROS are sequestered before they can cause any widespread damage. GPx mimics, in contrast, react directly and hence are more focussed. Such mimics primarily employ ROS on the one side and thiols, and occasionally some selenols, on the other, as their two substrates. This unique combination of sensing—of ROS—with the effects—on thiols—ensures a maximum of activity and selectivity, even at the molecular level. With this concept of effective yet selective catalytically active “sensor/effector” agents in mind, a colourful palette of compounds has been produced since the turn of the Millennium, with hues of pink for simple selenides and diselenides during early in proof-of-concept phases to considerably more complex structures with added features and activities, and often featuring the more active tellurium instead of selenium [72,83,84]. Some of this selenium chemistry has been highlighted in the review by Domínguez-Álvarez and his colleagues which forms part of this Special Issue.

In 2003, a small series of molecules combining a chalcogen with an additional quinone redox centre for extra activity has been reported, and the activity of these multifunctional redox modulating molecules in submicromolar concentrations and in cell lines such PC 12 by far eclipsed the one of simpler chalcogen-based GPx mimics considered under similar conditions a few years earlier [85,86]. Since then, the idea of multifunctional redox agents with ROS generating as well as ROS utilizing functionalities has inspired the development of a wider range of extraordinarily catalytic agents with two or more—mostly catalytic—redox sites brought together in one molecule. These agents have been evaluated rather successfully against a range of targets, from bacteria and fungi to cancer cells, macrophages and fibroblasts [87,88]. One particularly intriguing study considered the impact of these catalysts on chronic lymphoblastic leukaemia (CLL) cells derived from patients suffering from chronic lymphoblastic leukaemia in comparison to normal blood cells derived from the same, individual patients [89]. This study revealed a significant, pre-existing difference in levels of ROS between the cancer and normal cells. It also confirmed the ability of the tellurium catalyst employed to increase these ROS levels particularly strongly in the CLL cells but not in the normal cells, eventually pushing the cancer cells over a critical “redox threshold”. As expected, the induction of apoptosis was more significant in the cancer cells, and much lower in the normal cells, pointing towards a good selectivity of the catalyst and the underlying catalytic principle.

**Figure 5:**
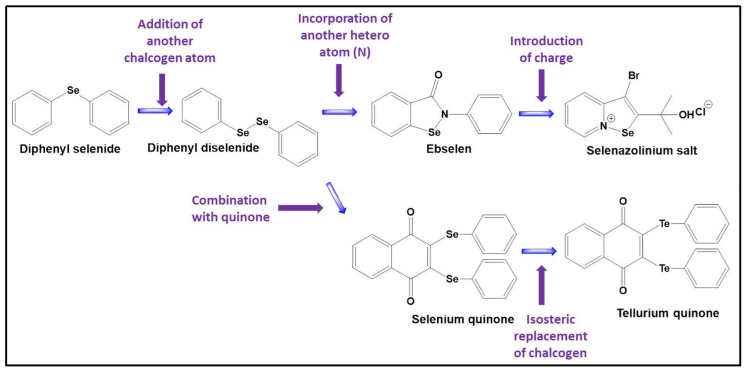
During the last couple of years, various strategies have been explored in order to increase the catalytic activity and selectivity of redox catalysts. See text for details.

Since then, this area of redox research has turned into a true El Dorado for synthetic chemists as well as biologists. From the perspective of chemistry, there are many ways to perfect such biologically active redox catalysts, from assembling complicated molecules with a well-tuned orchestra of multiple redox sites to keeping it small and replacing selenium with tellurium or fiddling with the selenium carburettor until the molecule gathers speed (Figure 5).

Eloquent coupling methods have been explored, for instance, to assemble multifunctional, multi-catalytic agents, from classical coupling to Passerini and Ugi multicomponent reactions [90]. Recently, Silva and his colleagues have used click chemistry to access a wide palette of such compounds combining quinones, including naturally occurring lapachones, with sulfur and selenium redox centres [91]. These and similar compounds have been evaluated rather successfully against a range of targets and may bear some promise in the field of antimicrobial, anti-inflammatory, scleroderma, psoriasis and anticancer research to name a few. Indeed, skin diseases may be a prime target for such compounds, as the research of Mueller and colleagues has indicated [92,93,94].

Whilst multi-catalytic agents may be considered as the El Dorado, tellurium is not only the name of a ghost town in Colorado, but far from being abandoned, also an interesting element to increase further activity and possibly also selectivity in chalcogen-based redox catalysts attacking the cellular thiolstat [95]. Within the niche of biological tellurium chemistry, we find quite a few compounds which act as potent, cell type-specific cytotoxic agents and hence may be employed against oxidatively stressed cancer cells [62]. Related compounds are rather selectively active against proliferating fibroblasts in in vitro and in mouse models of systemic sclerosis (scleroderma, SSc) [96]. It should be noted that tellurium, as a semi-metal, also forms some complex-like structures, such as compound AS101, which in many ways resemble some of the transition metal complexes mentioned above. Tellurium complexes frequently show some high, albeit not entirely well-behaved biological activity against target cells, yet with a possible negative impact on neurons [97].

The reactivity of selenium in compounds, for instance in the isoselenazole ebselen, can also be improved considerably by placing a positive charge at the nitrogen in the selenium-nitrogen bond. As Arsenyans and his colleagues have shown, the resulting selenazolinium salts are incredibly reactive and also active against a wide range of cancer cell lines [98]. Similarly, Handzlik and her colleagues, employing the same palette of selenazolinium salts have demonstrated that such compounds also act against certain resistant strains of bacteria [59]. Other unusual selenium compounds, for instance inspired by natural antioxidants, such as resveratrol, may be obtained by isosteric replacement of the omnipresent oxygen atoms in these natural products. Interestingly, certain selenium derivatives of resveratrol, i.e., benzo*[b]*selenophenes, protect the yeast *S. cerevisiae* from oxidative damage by decreasing the ROS level down to just 12% when compared to the control whereas resveratrol itself decreases the ROS level to 50% [99].

Besides these natural compounds with isosterically introduced selenium, there are many compounds which per se may be classified as “Reactive Selenium Species” (RSeS), as redox and biologically active selenium compounds of natural origin [100]. Albeit more limited in abundance when compared to the related class of “Reactive Sulfur Species” (RSS), these RSeS are also highly reactive, often catalytic and quite selective for thiols as one preferred cellular reaction partner and substrate, the other one being ROS. From a bio-catalytic perspective, selenols seem to be particularly interesting due to their catalytic activity, and whilst free selenols (RSeH) are incredibly sensitive towards oxidation, they can also be protected easily in form of diselenides or selenoesters. These esters seem to hydrolyse slowly, and the resulting selenium species appear to be rather reactive and also active in several biological test systems [101,102]. Recent studies by Sanmartin, Domínguez-Álvarez and their respective colleagues have confirmed this selenium “prodrug” approach employing esters, anhydrides and slow hydrolysis as a mean to stabilize and deliver highly active selenium species. In the future, researchers digging for the ultimate selenium catalysts may also revisit some of the already known reactive selenides and seleninic acids, including the lode of heterocyclic organic agents laid bare by Kirsch and his colleagues [103,104]. They may also turn towards small molecule catalysts attached to proteins, i.e., so-called “catalyst protein conjugates”, to ensure “delivery” or to biologically active nanoparticles composed entirely of catalytic material or composition of materials [105].

## 5. Targets for Redox Catalysts: A Selected Many

Traditionally, virtually all studies concerned with the synthesis of redox modulators, pro- and antioxidants alike, have also reported some information related to the redox activity and catalytic behaviour of such compounds in vitro, for instance in radical scavenging assays, specific glutathione peroxidase assays, dye-based redox assays or electrochemical studies [106,107,108,109,110]. Nowadays, an increasing number of publications also provides evidence in cell culture, ranging from bacteria, fungi and yeasts to mammalian cells. It is therefore hardly surprising that the question of cellular targets for these catalysts has emerged, a question which may not simply be answered by in vitro assays which certainly are instructive yet clearly fall short of the complexity of a living cell or even organism. In other words, a transition metal complex which may be catalytically active in the 96-multi-well plate may not exhibit the same activity in a multicellular organism, as it may be unable to enter cells, may not be stable under those conditions, may be sequestered by certain cellular components or may be metabolized and eventually kicked out of the back door before being able to exert its activity.

As a consequence, simple in vitro redox assays may be sufficient to demonstrate that certain antioxidants protect your apple juice from turning brown or—as mentioned in the Section 3—protect foodstuffs from oxidative degradation. Yet they are inadequate to predict or even explain the activity of redox catalysts in cells or entire organisms. To illuminate such mechanisms and modes of action, one needs to investigate living cells and small organisms with methods which enable analysis inside intact cells. Today, several, often complimentary approaches and techniques are available for this purpose, which include various proteomic methods, chemogenetic phenotype profiling, cell sorting and extensive process-selective fluorescent staining techniques known as “intracellular diagnostics” [87].

Redox proteomics has developed considerably during the last decade, from early “hunts” for posttranslational modifications in proteins to an extensive “mining” using eloquent and often expensive tools. These hunter-gatherer and miner studies have eventually surfaced a number of potential targets for redox modulation, among them many cysteine residues in proteins and enzymes, and have given rise to new concepts such as the “sulfenome” and the “redoxome” [111,112]. Compared to redox genomics, proteomics and metabolomics, chemogenetic phenotype profiling is perhaps not that well known [113]. It essentially screens single-mutant libraries, usually of *Saccharomyces cerevisiae*, for increased or decreased sensitivity towards a given agent and subsequently tries to associate the proteins affected by the mutation to the agent and its mode(s) of action. In contrast, to proteomics, which considers changes inside single cells in a more holistic manner and chemogenetic phenotype profiling, which in parallel looks at tens, hundreds or even thousands of mutants, “intracellular diagnostics” considers individual changes in individual cells employing specific fluorescent staining and visualization techniques. Importantly, such analysis can provide quantitative information crudely averaged over a range of cells using a fluorescent plate reader, quantitative information on the level of single cells using fluorescent assisted cell sorting (FACS) and even qualitative insights into cellular processes at different cellular locations within single cells using fluorescent microscopy. Amazingly, modern instruments are equipped with different lasers and it is therefore even possible to illuminate simultaneously several different cellular components and processes in the same sample or cell using different stains with distinct excitation and emission wavelengths. This approach relies heavily on the availability of selective stains, and since the turn of the Millennium, a wide spectrum of selective fluorescent stains has become available. Today, it is possible, for instance, to stain for—quantitative changes to—intracellular levels of various metal ions, ROS, singlet oxygen (^1^O_2_), the superoxide radical anion (O_2_^●−^), thiol levels, the mitochondrial membrane potential, caspase activity and phases of apoptosis, to name just a few.

During the last decade, such methods have been employed increasingly to decipher the mode(s) of action associated with many redox modulating and catalytic agents, including various selenols, selenides, seleninic acids and organotellurium compounds [62,88,96,114,115]. These studies have consistently revealed a considerable redox imbalance in typical target cells, such as macrophages, bacteria, plasmodia, yeasts and cancer cells. This pre-existing disturbance singles out the target from the healthy cells and, as may be expected, is usually potentiated by the redox catalysts, with a loss of cellular thiols and an increase in levels of ROS. Such changes do not go unnoticed by the cell, and whilst some cells, such as retinal endothelial ARPE 19 cells, eventually mount an antioxidant response via factors such as Nrf2, other cells, such as human cancer HCT 116 cells, under the same conditions enter into apoptosis [116,117,118]. Interestingly, “intracellular diagnostics” has also revealed some striking differences between the action of redox catalysts on the one side and traditional cytotoxic agents and inducers on the other. First of all, the catalysts are not selective for just one protein, for instance, one particular receptor or enzyme, and hence differ from the kind of classic inhibitors mentioned in the Introduction. Similar to the cytotoxic transition metal complexes discussed in Section 3, such quinone and chalcogen-based catalysts are rather indiscriminate and simultaneously target numerous proteins as their perceived substrate(s), usually but not always particularly redox sensitive cysteine proteins with cysteine accessible for modification, such as *β*-tubulin [119]. Yet in clear contrast to the noble metal catalysts developed by Sadler and colleagues at Warwick, chalcogen-based catalysts do not simply deplete the cell of its fuel or GSH, or poison the cell with ROS or unnatural products. Such catalysts operate rather via the oxidation of thiol and selenol proteins, and as such trigger well established cellular response cascades which eventually lead to a recovery or apoptosis. This rather unusual specificity for certain cysteine proteins under certain conditions of pre-existing oxidative stress eventually explains, at least in part, the considerable yet selective activity observed in certain target cells, such as plasmodia, bacteria, macrophages, cancer cells and even for uncontrolled proliferating fibroblasts in a mouse model of SSc [96,120].

As a biological twist, such studies have also revealed that not all cysteine peptides, proteins and enzymes are equally prone to oxidation, as one may have anticipated naïvely along the lines of “cysteine being cysteine”. GSH, for instance, has long been thought to protect protein thiols from oxidation because of its sheer abundance, often reaching millimolar concentrations in the cytosol. Yet GSH is surprisingly sluggish in its reactivity and some proteins, such as *β*-tubulin, and also certain enzymes, despite their comparably low abundance, seem to become oxidatively modified even in the presence of a large excess of GSH. This surprising sensitivity is probably due to a combination of thermodynamic and kinetic factors, such as electrochemical redox potential, exposure on the surface, cellular compartmental location and the kinetics of the reaction. Indeed, it seems that only a limited number of cysteine proteins respond initially or extensively to an oxidative onslaught, and in a measured and largely reversible manner. Even a more substantial external redox attack often proceeds in a rather well-defined manner. Cellular proteins, rather than the catalyst itself, provide a certain selectivity here, of target and also of response, a finding which has given rise to the concept of the “cellular thiolstat” [65]. The “cellular thiolstat” brings together the most reactive, yet otherwise diverse cysteine proteins and enzymes as the prime targets for redox modulation, sensing, response and signalling, thereby providing a rationale for the action of redox modulating and catalytic agents. It should be mentioned that the concept of the “cellular thiolstat” is constantly developing. These days, additional proteins are being identified as being modified by different redox active agents and able to convey cellular signals, and these signals are also being studied in more detail.

## 6. Conclusions and Future Perspectives

The previous sections have highlighted some of the rather unique features of catalytic agents in drug development, from the unique combination of high efficiency and selectivity of therapeutically administered enzymes to the ability of small molecule catalysts to initiate, promote, enhance or even redirect energetically favourable reactions. Certain drawbacks, such as poor bioavailability, chemical and metabolic instability and shocking immune responses against therapeutic proteins have also been mentioned and no doubt complicate the development and applications of such molecules. Still, the success of very different catalysts, which range from osmium complexes catalysing transfer hydrogenation, lactate formation from formate and reductive stress on one side to organotellurium compounds facilitating an oxidative onslaught on redox sensitive proteins of the “cellular thiolstat” in the presence of elevated levels of ROS on the other, demonstrates that the catalytic principle is not simply an idée fixe limited to one or two exotic compounds. In fact, redox-active small molecule catalysts—and among them mimics of specific redox enzymes such as SOD and GPx in particular—act in low concentrations and with considerable selectively, depending on the presence of their substrates. These catalysts are superior to their parent enzymes, as they do not carry any “protein ballast” and are also more flexible in their design, physico-chemical properties and applications.

Future studies in this field of biological redox catalysis are therefore promising and supported by the fact that one catalytic selenium compound, namely ebselen, has already reached clinical trials. Similar RSeS, far from being “alien” to biology, are found increasingly naturally in humans and animals, one only needs to consider the selenium analogue of ergothioneine, namely selenoneine, which was recently discovered in the blood of tuna fish [121]. This chemically unusual selenone is probably only the tip of the iceberg of not yet identified natural RSeS in Biology, including in mammals and in humans.

Fortunately, the development of such redox catalysts can rely on strong support from analytical and synthetic chemistry, with an increasing number of researchers worldwide joining the effort to design, develop and test such catalysts against a wide range of human diseases, from cancer and infections to inflammation and scleroderma. Ironically, the chemistry of elements such as selenium and tellurium, iridium and osmium, long confined to niches and of limited interest in biology—where quite a few students still believe that selenium and osmium are homage on Selena Gomez and Ozzie Osbourne—is bearing significant promise. A recent renaissance of selenium chemistry with meetings such as the BioSePe meeting 2016 in Cracow reflects the growing interest in this field, and one may anticipate similar developments in the years to come. In the future, interest may not only revolve around traditional catalysts “borrowed” from chemistry and “dumped” into biological systems, such as the initial generations of manganese and other transition metal complexes, but rather focus on well selected and designed agents. No doubt, these catalysts will still be inspired by existing knowledge of chemical catalysis, yet will also adhere to basic principles of biochemistry and drug development.

Within such a multidisciplinary ménage à trois of synthetic chemistry, biochemistry and drug development, future research may follow several interesting leads. The prospect of assembling multifunctional redox modulators which perform several tasks, such as ROS generation via a quinone and catalytic ROS conversion(s) via a selenium or tellurium moiety may be expanded to a more sophisticated kind of mimics which no longer imitates the function of an individual enzyme, but of an entire (immune) cell. From the perspective of (cell) biology, the idea of a partially synthetic “immune cell mimic” is, of course, a daring, challenging idea, but also one with considerable promise. From the perspective of chemistry, all one can say is “yes, we can”, as combinations of quinones, chalcogens and transition metal ions, perhaps even in macromolecules or in nanoparticles, are not out of reach. Indeed, whilst a simple selenium substitution in a given flavonoid such as quercetin adds a scent of redox catalysis, implementing such a change in an oligomeric, tannin-like proanthocyanidin results in a macromolecule with massive redox firing power based on multiple quinone and chalcogen redox centres. Similarly, artificial proteins rich in selenocysteine residues, possibly stabilized by complexing redox active metal ions, or polymeric selenides, seleninic acids or selenocysteine, would also be of considerable interest. At the same time, one may envisage small molecule catalysts attached to carrier proteins, similar to the alliinase-antibody hybrids [105]. Or why not go “solid” by considering catalytic nanomaterials, such as synthetic or even natural nanoparticles of sulfur, selenium or tellurium? [122,123,124,125].

No doubt, synthetic chemistry will be at the forefront of this research, it will have to provide the means to “assemble” the various required features in comparably small molecule catalysts amenable for potential pharmaceutical applications. Mode of action studies, which will have to accompany any activity screens, will provide further evidence as to which cells are particularly sensitive to redox regulation and how exactly they are affected by redox modulation. At the same time, most of the compounds discussed here have not yet been investigated thoroughly for their toxicity, medium and long-term side effects and metabolic transformation in more complex organisms. Here, bioavailability will become an issue, as some of the agents employed so far are only poorly soluble and may need to be nanosized and stabilized as part of the NaLyRe sequence in order to be applied [122]. Whilst data already available in some mammals appears promising, clinical trials in this field so far have almost solely focussed on the SOD mimic manganofodipir and the GPx mimic ebselen.

Irrespective of such “hopes, aspirations and telephone numbers”—to quote Bert L. Vallee—and one may add “concerns”, the features of catalysis, which are deeply rooted in biology, are extraordinarily attractive and impossible to ignore in the field of drug design. With many human, animal and plant diseases on the rise and new and innovative agents desperately required, why not give the large and small molecule catalysts a genuine chance?

## Figures and Tables

**Table 1:** Enzymes and enzyme mimics may both be employed in prevention and therapy and both provide individual advantages and disadvantages. Hybrid molecules have recently been considered as a mean to combine such benefits whilst minimizing any drawbacks. See text for details.

Parameters	Enzyme (±Substrate)	Small Molecule Catalyst (Mimic)	Hybrid of Small Molecule Catalyst and Protein
Origin	Natural	Artificial	Combination
Molecular weight	High (well above 10 to 100 kDa)	Low (100–300 Da)	High
Stability	Sensitive to degradation	Generally stable	Intermediate
Administration	Usually intravenously	Oral administration feasible	Usually intravenously
Immune response	Common if administered intravenously	Rare	Possible but avoidable
Selectivity for substrates	Highly selective for specific substrates	May use various substrates	May use various substrates
Selectivity for pre-existing biochemical signatures	Can be selective if substrate present	Can be selective if substrate present	Can be selective if substrate present
Active transport to cellular targets	Usually not selective	Not selective	Possible if antibodies are involved
